# Age- and sex-associated alterations in hypothalamic mitochondrial bioenergetics and inflammatory-associated signaling in the 3xTg mouse model of Alzheimer’s disease

**DOI:** 10.1186/s13293-024-00671-7

**Published:** 2024-11-25

**Authors:** Aida Adlimoghaddam, Kyle M. Fontaine, Benedict C. Albensi

**Affiliations:** 1 Department of Neurology, Dale and Deborah Smith Center for Alzheimer’s Research and Treatment, Neuroscience Institute, Southern Illinois University School of Medicine, Springfield, IL USA; 2 Department of Pharmacology, Southern Illinois University School of Medicine, Springfield, IL USA; 3 Department of Medical Microbiology, Immunology and Cell Biology, Southern Illinois University School of Medicine, Springfield, IL USA; 4 Department of Pharmaceutical Sciences, Barry & Judy Silverman College of Pharmacy, Nova Southeastern University, Fort Lauderdale, FL USA; 5 Division of Neurodegenerative Disorders, St. Boniface Hospital Albrechtsen Research Centre, Winnipeg, MB Canada; 6 Department of Pharmacology & Therapeutics, Max Rady College of Medicine, University of Manitoba, Winnipeg, MB, Canada

**Keywords:** Alzheimer’s disease, 3xTg, Hypothalamus, NF-κB, Nrf2, Bioenergetic profiling, Mitochondria, Inflammation

## Abstract

Mitochondrial dysfunction and associated inflammatory signaling are pivotal in both aging and in Alzheimer’s disease (AD). Studies have also shown that hypothalamic function is affected in AD. The hypothalamus may be a target for AD drugs given that mitochondrial alterations are observed in the hypothalamus. This study investigated how age and sex affect mitochondrial bioenergetics and inflammatory signaling in the hypothalamic mitochondria of 3xTg and control mice at 2, 6, and 13 months, aiming to enhance our understanding of these processes in aging and AD. Parameters included oxygen consumption rates, expression levels of subunits comprising mitochondrial complexes I-V, the enzymatic activity of cytochrome c oxidase (COX), transcription factors associated with inflammation such as NF-κB, pIκB-α, Nrf2, and other inflammatory biomarkers. Hypothalamic mitochondrial dysfunction was observed in 3xTg females as early as 2 months, but no changes were detected in 3xTg males until 6 months of age. In 3xTg mice, subunit expression levels for mitochondrial complexes I-II were significantly reduced in both sexes. Significant sex-based differences in COX activity were also observed at 13 months of age, with levels being lower in females compared to males. In addition, significant sex differences were indicated in NF-κB, pIκB-α, Nrf2, and other inflammatory biomarkers at different age groups during normal aging and AD progression. These findings highlight important sex differences in hypothalamic bioenergetics and inflammation, offering insights into potential new targets for preventing and/or treating AD.

## Abstract (plain language summary)

Alzheimer’s disease (AD) is a gradual onset disorder, characterized by memory impairments. The greatest risk factor is age. In addition, almost two-thirds of Americans with AD are women, and reasons for this health disparity are unclear. While it’s true that women live longer than men, longevity alone does not explain this disparity. Interestingly, studies have also shown that hypothalamic function is affected in AD, but it appears that men are affected differently than women. The hypothalamus regulates numerous functions, including sex hormone regulation, sleep/wake cycles, and energy expenditure, to name a few. In this study, we examined how changes in energy metabolism may be different in the hypothalamus in male vs. female genetically engineered mice that model AD pathology.

## Highlights

Hypothalamic mitochondrial dysfunction was observed in 3xTg females as early as 2 months, but no changes were detected in 3xTg males until 6 months of age.

In 3xTg mice, sex-based differences were observed in the expression levels of subunits for mitochondrial complexes II and III.

Significant sex-based differences in COX activity were also observed at 13 months of age, with levels being lower in females compared to males.

Significant sex differences were indicated in NF-κB, pIκB-α, Nrf2, and other inflammatory biomarkers at different age groups during normal aging and AD progression.

## Introduction

Alzheimer’s disease (AD) is a gradual onset, multifactorial, neurodegenerative disorder, characterized by progressive cognitive impairments [1, 2]. Emerging evidence now demonstrates that the earliest deficits in the pathological progression of AD are associated with impaired energy metabolism [3]. Energy metabolism is regulated by the central nervous system (CNS) [4]. Within the CNS, in particular, the hypothalamus regulates numerous metabolic functions, including energy expenditure [5], sex hormone regulation [6] sleep/wake cycles [7], and circadian rhythms [8], to name a few. Neuroimaging research suggests that atrophy in the hypothalamus, marked by a decrease in volume, is linked to AD pathology (Aβ42, tau, and phosphorylated tau) and signals structural damage in this vital brain area, underscoring its significance in the disease’s progression [9, 10]. Previous studies have demonstrated that the total cell population in the suprachiasmatic nucleus (SCN), located in the hypothalamus, declines with aging and is significantly reduced in AD [11, 12]. The decrease in SCN cell numbers may be linked to functional differences among the hypothalamic nuclei in AD [13]. Other studies using immunohistochemistry in AD brains confirmed the presence of neuronal loss, in the hypothalamus [9]. Also, abnormalities in hypothalamic metabolism and cerebral blood flow have been observed in the APP/PS1 mouse model of AD, occurring before the onset of cognitive decline [14]. In the Tg2476 mouse model of AD, neurons producing neuropeptide Y (NPY) neurons in the hypothalamus exhibited reduced responsiveness to appetite-regulating hormones leptin and ghrelin, a change also seen with Aβ treatment in normal mice [9, 15]. The interplay between hypothalamic dysfunction and AD pathology underscores the complex relationship between metabolic regulation, hormonal signaling, and neurodegenerative disease [16]. Interestingly, alterations in brain bioenergetics also intersect with inflammatory processes [17] and the hypothalamus is involved in regulating the body’s response to inflammation due to its role in regulating the immune system and coordinating various physiological processes [18]. The inflammatory responses vary between males and females [19], and sex differences exist in the prevalence of diseases, however; sex- and age-associated hypothalamic inflammation in AD is not completely understood. Emerging evidence suggests that nuclear factor kappa B (NF-κB), which is a master switch and central regulator of inflammatory processes, is also functioning as a critical regulator of energy homeostasis and metabolic adaptation through upregulating mitochondrial respiration metabolism [20–24]. Another study found that the NF-κB signaling pathway can negatively regulate mitochondrial Complex IV which is associated with mitochondrial oxygen consumption [25, 26]. However, whether there are hypothalamic NF-κB-associated alterations during aging and the progression of AD remains unexplored. The consequence of sex-based differences on hypothalamic NF-κB levels in AD also has not been examined.

Nuclear factor erythroid 2-related factor 2 (Nrf2) is another key transcription factor that regulates oxidative stress that drives age-related pathologies [27]. In addition, it has been reported that Nrf2 declines with age and Nrf2 deficiency leads to mitochondrial dysfunction [28]. However, sex-specific transcriptional regulation of Nrf2 during normal aging and AD has not been studied completely.

Emerging evidence has also emphasized the potential of pro-inflammatory cytokines such as interleukin-1β (IL- β), tumor necrosis factor-α (TNF-α), and interleukin-6 (IL-6), as biomarkers for AD [29]. Interestingly, these cytokines are also influenced by the female sex hormone estrogen, which suppresses inflammation at high levels and promotes it at low levels [19]. Therefore, focusing on inflammatory markers provides several mechanistic approaches to better comprehend sex-based differences in AD. Further, cytokines known as interferons exhibit a major role in AD pathology [30]. For instance, pre-treatment of human neuroblastoma cells with interferon (IFN-γ) significantly increased the sensitivity of neurons to the toxic effects of amyloid beta (Aβ) [31]. This suggests that IFN-γ may enhance neuronal degeneration in AD. Other studies observed an association between elevated IFN-γ levels and reduced cognitive decline, independent of Aβ levels [30, 32]. Additionally, higher IL-12p70 levels were linked with reduced tau pathology and neurodegeneration in participants with higher Aβ [33]. Monocyte chemoattractant protein-1 (MCP-1) is one of the key chemokines that mediate inflammation in AD [34]. A two-year clinical study indicated that greater severity and faster cognitive decline in AD are associated with higher levels of plasma MCP-1 [35]. Other cytokines and inflammatory markers such as IL-2, IL-4, Il-17 A, and IL-10 are major cytokines associated with the occurrence of AD [36–39]. However, sex- and age-related inflammatory changes in the hypothalamus have not been investigated.

In this study, we measured the age- and sex-associated differences in mitochondrial bioenergetic profiling and inflammatory-associated signaling in hypothalamic mitochondria from 3xTg and their controls at 2, 6, and 13 months of age. Parameters measured included mitochondrial oxygen consumption rates (OCR), alterations in OXPHOS, cytochrome c oxidase (COX) activity, transcription factors such as NF-κB and its associated subunits (NF-κB p50, p105, p65, p75), pIκB-α, Nrf2, inflammatory biomarkers such as TNF-α, IFN-γ, IL-1α, IL- β, IL-2, IL-4, IL-5, IL-6, IL-7, IL-10, IL-12p70, IL-13, IL-17 A, MCP-1, MIP-2, KC, LIX, and GM-CSF were evaluated in both males and females in 3xTg mice vs. age-matched male and female control mice at 2, 6, and 13 months old.

## Materials and methods

### Animals

In this study, we employed the triple-transgenic (3xTg #033930-JAX; Jackson Laboratories; Bar Harbor, Maine) mouse model along with the C57BL/6J-congenic background [40, 41]. The 3xTg mouse model of AD develops age-related cognitive impairments and amyloid-beta (Aβ) pathology by 6 months of age [33]. By 12 months, it also develops neuritic plaques and neurofibrillary tau tangles. To investigate the effect of age and sex-associated differences in mitochondrial deficits and also NFκB signaling within the hypothalamus, a total of 60 mice (2 strains of animals (3xTg andC57BL/6) X 2 sexes of animals (males and females) X 5 biological replicates = 60) were utilized. Both male and female 3xTg (AD) and C57BL/6 (control) mice were euthanized at 2 months, 6 months, and 13 months of age. Hypothalamic tissue was then dissected from the brain following decapitation. All animal procedures followed the guidelines of the Institutional Animal Care and Use Committee (IACUC) standards. Groups of mice were given *ad libitum* access to food and water and housed on a 12-hour light/12-h dark schedule and 40% humidity.

### Preparation of isolated mitochondria from hypothalamic tissue

The mice were humanely euthanized by performing decapitation, which was conducted while they were under isoflurane anesthesia. Hypothalamic tissues were then removed. Mitochondria derived from both 3xTg and control mice were extracted using specialized commercial isolation kits (110170, Abcam, Cambridge, Massachusetts, USA) as reported previously [24, 26, 42]. Briefly, tissues were initially rinsed with 1x Phosphate-buffered saline (PBS). Following this, they were homogenized in a mitochondrial isolation buffer (MIP) designed to preserve mitochondrial integrity. The resulting homogenates underwent centrifugation at 1,000 × g for 5 min at a temperature of 4 °C. Then supernatants obtained from this step were then subjected to a second round of centrifugation at 10,000 × *g* for 20 min at 4 °C. Pellets to isolate the mitochondrial fraction). The resultant pellets, which contained the isolated mitochondria, were resuspended in MIP Subsequently, the protein concentrations in theses samples were determined using a colorimetric DC protein assay kit (BioRad, Hercules, California, USA) following the protocol described previously [43].

### Measurement of mitochondrial respiration rates

Real-time measurements of oxygen consumption rates (OCR) were conducted on the isolated hypothalamic mitochondria employing the Seahorse XF Analyzer (Agilent Technologies, Santa Clara, CA, USA) [24, 26, 44]. To assess mitochondrial respiration, freshly isolated mitochondrial protein (15 µg/well) was prepared in mitochondrial assay solution (MAS). This solution comprised 70 mM sucrose, 220 mM mannitol, 10 mM KH2PO4, 5 mM MgCl2, 2 mM HEPES, 1 mM EGTA, and 0.2% BSA along with 2 mM malate and 10 mM pyruvate. The prepared mixture was then carefully transferred onto a Bioscience plate for analysis. Initial measurements of basal respiration were measured in the presence of malate (2 mM) and pyruvate (10 mM). Following this, coupled respiration was assessed by adding adenosine-diphosphate (ADP) at a concentration of 4 mM, which serves as an ATP synthase substrate). Subsequently, to evaluate coupled respiration ligomycin (2 µM), an ATP synthase inhibitor was then added. To evaluate maximal respiration, the protonophore carbonyl cyanide p-triflouromethoxy-phenylhydrazone (FCCP) as a respiratory chain uncoupler was added at 4 µM. To complete the analysis, rotenone (2 µM) as a specific inhibitor of complex I, and antimycin A (2 µM) which inhibits complex III were added to effectively block mitochondrial respiration. All OCR data were then analysed with subtracting the non-mitochondrial respiration values. OCR readings were captured and analyzed using the Seahorse XF-24 software (Seahorse Biosciences, Billerica, MA, USA). To ensure accuracy, the OCR level was normalized based on total protein concentrations (36). These protein concentrations were determined using the DC Protein Assay (Bio-Rad, Hercules, California, USA). The final protein quantification was performed utilizing the ChemiDocTM MP (Stain-FreeTM).

### Western blot analysis

Initially, hypothalamic tissues were mixed with 4X laemmli buffer and heated at 55 °C for eight minutes. Following this preparation, 15 ug per protein from each sample was carefully loaded into the wells of sodium dodecyl sulfate-polyacrylamide (SDS-PAGE) gels (Bio-Rad, Hercules, CA, USA). The proteins were separated by electrophoresis, which was conducted at 200 volts for a duration of 60 min. Once the electrophoresis was complete, the gels were activated using a ChemiDoc imaging system (Bio-Rad, Hercules, California, USA). Next, the proteins were transferred from gels into the nitrocellulose membranes (Bio-Rad, Hercules, California, USA) using the Trans-Blot Turbo Transfer System (Bio-Rad, Hercules, California, USA). Following a transfer, the total proteins present on the membranes were detected and visualized using the ChemiDoc imager. Following the imaging process, the nitrocellulose membranes were incubated in TBS-T buffer containing 5% bovine serum albumin (BSA) and incubated for one hour at room temperature to minimize non-specific binding. Subsequently, the membranes were treated with a blocking buffer, which consisted of 5% bovine serum albumin (BSA) dissolved in Tris-buffered saline with 0.1% Tween-20 (TBS-T). The membranes were immersed in this blocking buffer for one hour at room temperature to prevent any unintended bindings of antibodies to the membrane. Following blocking, the membranes were incubated with primary antibodies: Total OXPHOS Rodent WB Antibody Cocktail (ab110413, Abcam, Cambridge, Massachusetts, USA, 1:1000 dilution), NF-κB p50/p105 (ab32360, Abcam, Cambridge, UK; 1:1000), NF-κB p65 (ab16502, Abcam Cambridge, UK; 1:1000), NF-κB p75 (ab108299, Abcam, Cambridge, UK; 1:1000), and pIκB-α (cs2859, Cell Signaling, Danvers, USA; 1:500). Following primary antibody incubation, the membranes were thoroughly washed with 1X TBS-T buffer. This washing process involved three separate washes, each lasting 15 min, to ensure the removal of any unbound primary antibodies and reduce background signals. Following the washes, the membranes were then incubated with HRP-conjugated secondary antibodies (Jackson ImmunoResearch Laboratories, West Grove, PA, USA, 1:2000 dilution) prepared in TBS-T buffer containing 5% BSA for a duration of one hour at room temperature. Subsequent to the secondary antibody incubation, the membranes underwent another series of three washes with 1X TBS-T buffer each lasting 15 min. To visualize the protein bands, the membranes were treated with enhanced chemiluminescence (ECL) using the Bio-Rad Clarity Weston ECL blotting kit (Bio-Rad, Hercules, California, USA). The chemiluminescent signals were then captured and analyzed using the ChemiDoc. Finally, to assess the relative levels of proteins, the quantification was normalized to the total protein content as previously described [45].

### Measurement of cytochrome c oxidase activity

To evaluate the enzymatic activity of mitochondrial cytochrome c oxidase (COX) a spectrophotometric analysis was performed using Ultrospec 2100 pro; GE Healthcare. This assessment was conducted on mitochondrial preparations isolated from the hypothalamus and utilized an assay kit provided by Abcam (ab239711) The specific activity of COX was assessed using a colorimetric method. The involved measuring the decrease in absorbance at 550 nm, which reflects the oxidation of reduced cytochrome c, as detailed in prior studies [24, 26, 42].

### Multiplex cytokines analysis

Hypothalamic tissues were harvested at three different time points (2, 6, and 13 months) from 3xTg and C57 mice and then stored at − 80 °C. Further, tissues were homogenized in PBS with protease and phosphatase inhibitors. Homogenates were centrifuged at 10,000 × *g* for 10 min at 4 °C. Next, the protein concentrations of samples were measured using a colorimetric DC protein assay kit (Bio-Rad, Hercules, California, USA). Multiplex assay was performed at Eve Technologies by using the Bio-Plex 200 system (Bio-Rad Laboratories, Inc.), and a Milliplex Mouse Cytokine/Chemokine kit (Millipore) according to their protocol [46]. The 18-plex panel consisted of TNF-α, IFN-γ, IL-1α, IL- β, IL-2, IL-4, IL-5, IL-6, IL-7, IL-10, IL-12p70, IL-13, IL-17 A, MCP-1, MIP-2, KC, LIX, and GM-CSF. All samples were analyzed in triplicates. The standard curve regression was used to calculate the concentration of each target cytokine.

### Statistical analysis

We used R version 4.4.1 (R Core Team 2024) and the following R packages along with the following R packages to conduct a full functional three-way ANOVA (age x genotype x sex): easystats v. 0.7.2 (Lüdecke et al. 2022), export v. 0.3.0 (Wenseleers and Vanderaa 2022), ggpubr v. 0.6.0 (Kassambara 2023a), ggstatsplot v. 0.12.4 (Patil 2021), gt v. 0.10.1 (Iannone et al. 2024), openxlsx v. 4.2.5.2 (Schauberger and Walker 2023), quarto v. 1.4 (Allaire and Dervieux 2024), rmarkdown v. 2.27 (Xie, Allaire, and Grolemund 2018; Xie, Dervieux, and Riederer 2020; Allaire et al. 2024), rstatix v. 0.7.2 (Kassambara 2023b), shiny v. 1.8.1.1 (Chang et al. 2024), tidyverse v. 2.0.0 (Wickham et al. 2019). Some of the data were subjected to statistical analysis using a two-way ANOVA, with subsequent Tukey *post-hoc* tests (GraphPad Prism 9, GraphPad Software) to assess the statistical significance of differences between the various groups. The results are presented as mean ± standard deviation (SD) to reflect the variability within the data. A difference between or among groups was considered statistically significant if * *P* ≤ 0.05, **P ≤ 0.01, *** *P* ≤ 0.001, and ***P ≤ 0.001.

## Results

### Mitochondrial respiration rates

**Fig. 1:**
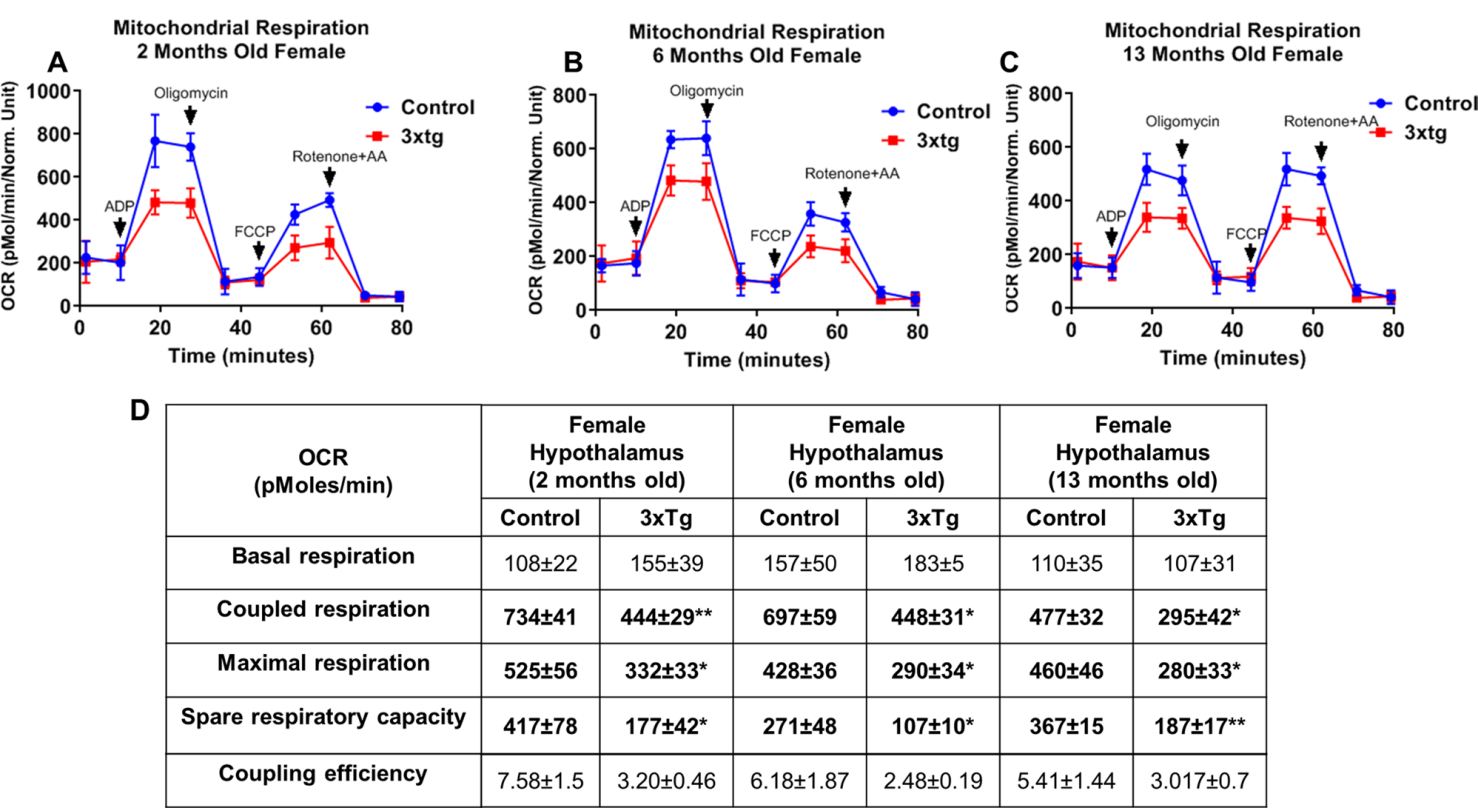
Oxygen consumption rates (OCR) in hypothalamic mitochondria, comparing 3xTg females with control females. Representative measurements of OCR in isolated hypothalamic mitochondria from 3xTg and age-matched control females at three different ages: 2 months (**A**), 6 months (**B**) and 13 months of age (**C**). The data included real-time kinetics, which were recorded at baseline and following the addition of ADP, oligomycin, FCCP, and rotenone/antimycin **A**. Table **D** provides a detailed summary of the bioenergetic parameters, including basal, coupled, maximal respiration, spare respiratory capacity, and coupling efficiency for both 3xTg and aged-matched control females at the ages of 2, 6 and 13 months. Significant reductions were observed in the coupled, maximal respiration, and spare respiratory capacity in the 3xTg females compared to the aged-matched control group at 6 and 13 months of age. The results are reported as OCR values in pmoles/min, with all data expressed as mean ± SD of *n* = 5 replicates (*P ≤ 0.05, **P ≤ 0.01) analyzed by two-way ANOVA

**Fig. 2:**
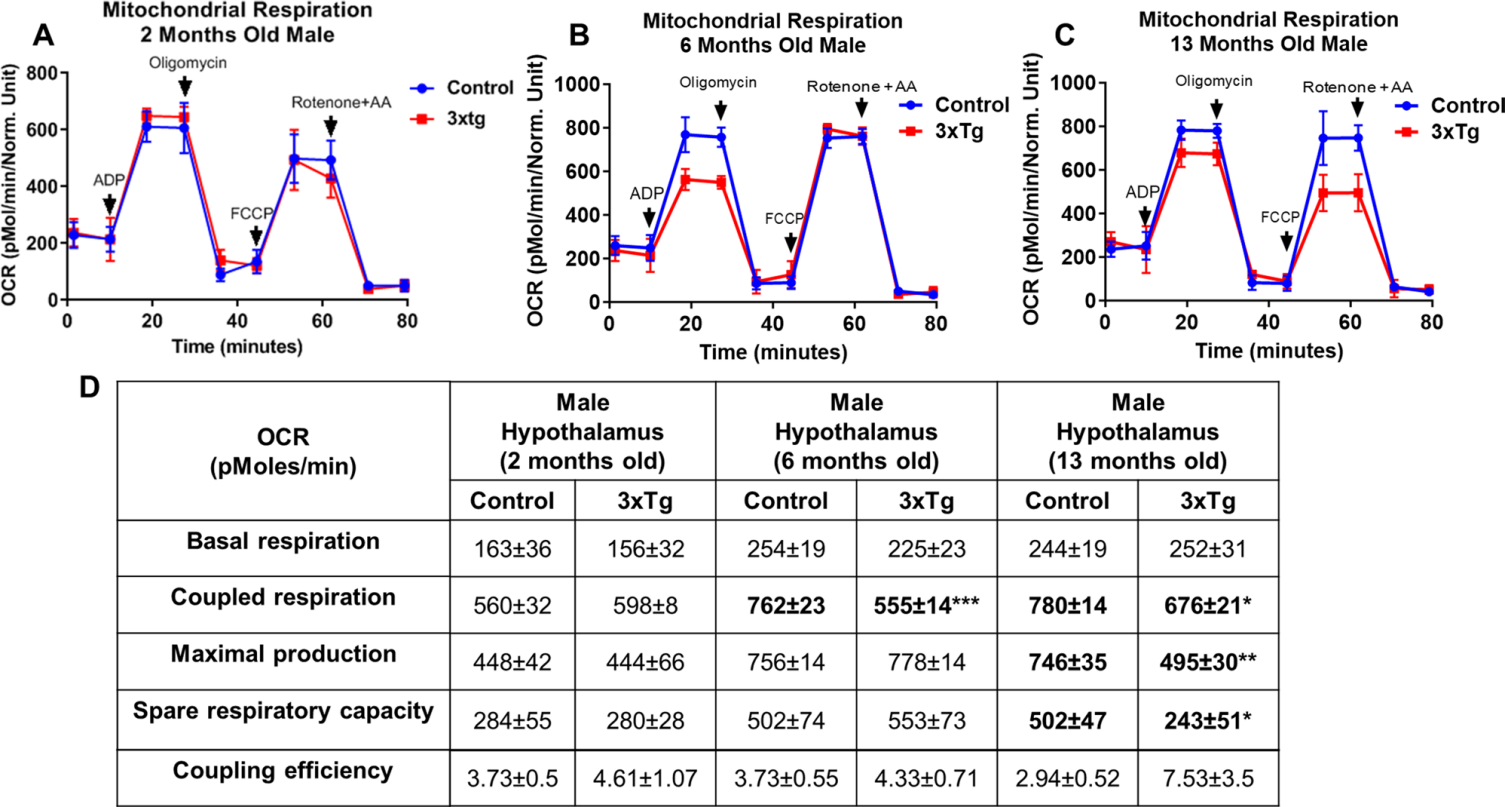
Oxygen consumption rates (OCR) in hypothalamic mitochondria, comparing 3xTg males with control males. Representative measurements of OCR in isolated hypothalamic mitochondria from 3xTg and age-matched control males at three different ages: 2 months (**A**), 6 months (**B**), and 13 months of age (**C**). Calculated parameters, which were recorded at baseline and following the addition of ADP, oligomycin, FCCP, and rotenone/antimycin **A**. Table **D** provides a detailed summary of the bioenergetic parameters, including basal, coupled, maximal respiration, spare respiratory capacity, and coupling efficiency for both 3xTg and aged-matched control males at the ages of 2, 6 and 13 months. No significant variations were found in the bioenergetic profiles across different ages or between the 3xTg and control strains. All data are expressed as OCR in pmoles/min, and values are expressed as mean ± SD of *n* = 5 replicates (*P ≤ 0.05, **P ≤ 0.01, and *** *P* ≤ 0.001) analyzed by two-way ANOVA

To assess alterations in bioenergetics, we conducted measurements of d OCR in freshly isolated mitochondria derived from the hypothalamus of female and male 3xTg at various age groups: 2, 6, and 13 months-old females (refer to Fig. 1A-D for females and Fig. 2A-D for males) alongside their respective controls. Our analysis revealed no significant differences in the baseline OCR of mitochondria from 3xTg mice across the different ages (2, 6, and 13 months). We observed a notable reduction in coupled respiration was significantly decreased in the hypothalamic tissues of 2-month 3xTg females as illustrated in Fig. 1B. This significant decrease in coupled respiration was maintained through subsequent ages, including 6 and 13 months old as shown in Fig. 1C). A significant decline in maximal respiration was observed in the hypothalamic mitochondria of 3xTg females at 2, 6, and 13 months of age when compared to their control counterparts (Fig. 1B and C). In addition to basal, coupled, and maximal respiration, we also assessed spare respiratory. The data revealed that spare respiratory capacity in female 3xTg mice was significantly reduced throughout the progression of AD at 2, 6, and 13 months old relative to age-matched control females (as shown in Fig. 1A-C). This reduction suggests that the hypothalamus in 3xTg females is less able to meet increased energy demands associated with disease progression. Despite these findings, no significant differences were detected in coupling efficiency defined as coupled respiration relative to basal levels- between 3xTg and control female mice (Table D). In 3xTg males, mitochondrial bioenergetics parameters at basal, coupled, and maximal respiration, as well as spare respiratory capacity levels, showed no significant changes at 2 months of age compared to control males (Fig. 2A). However, by 6 months, there was a significant decrease in coupled respiration. At 13 months, reductions were observed in coupled respiration, maximal respiration, and spare respiratory capacity compared to age-matched control males (Fig. 2B-C). All measurements are reported as OCR in pmoles/min, with values expressed as mean ± SD of *n* = 4–5 replicates. To further investigate molecular mechanisms potentially responsible for the diminished mitochondrial function in 3xTg, we conducted a Western blot analysis on various proteins in the hypothalamus.

### Mitochondrial proteins

**Fig. 3:**
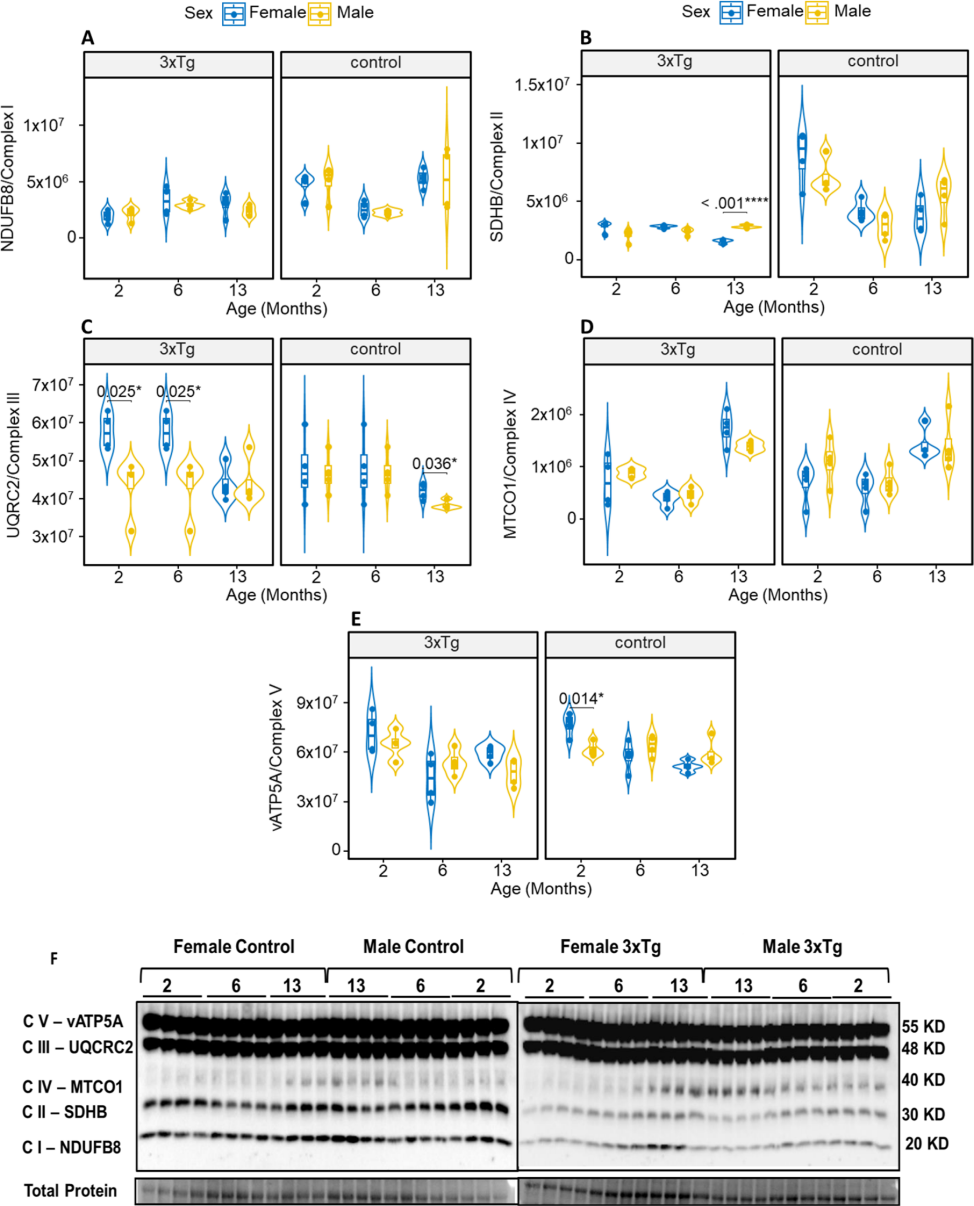
OXPHOS levels in hypothalamic mitochondria of 3xTg male and female mice compared to controls. Western blot images show representative bands for NADH dehydrogenase beta subcomplex subunit 8 of Complex I (NDUFB8; **A**), succinate dehydrogenase subunit B of complex II (SDHB; **B**), cytochrome b-c1 complex subunit 2 of complex III (UQCRC2; **C**), cytochrome c oxidase subunit 1 of complex IV (MTCO1; **D**), and ATP synthase subunit alpha of complex V (ATP5A; **E**). Quantification of these proteins, normalized to total protein (**F**) for 3xTg female and male mice and their respective controls at 2, 6, and 13 months of age. Asterisks indicate statistical significance compared to corresponding control groups. Results are presented as mean ± SD of *n* = 4 per group, with significance levels denoted as *P ≤ 0.05, **P ≤ 0.01, and ***P ≤ 0.001, analyzed using functional three-way ANOVA. In female 3xTg mice, complex II expression was significantly reduced compared to males, while no sex-based differences were observed in control mice across all age groups (**A**). Complex III levels were higher in female 3xTg mice at 2 and 6 months, whereas control mice showed an increase only at 13 months (**B**). No significant differences in complex V expression were noted in 3xTg mice; however, at 2 months, control mice exhibited higher levels in females, with no notable differences in other age groups (**C**). Additionally, there were no significant variations in the levels of protein subunits for complexes I and IV across all age groups, regardless of genotype or sex (**D**)

**Fig. 4:**
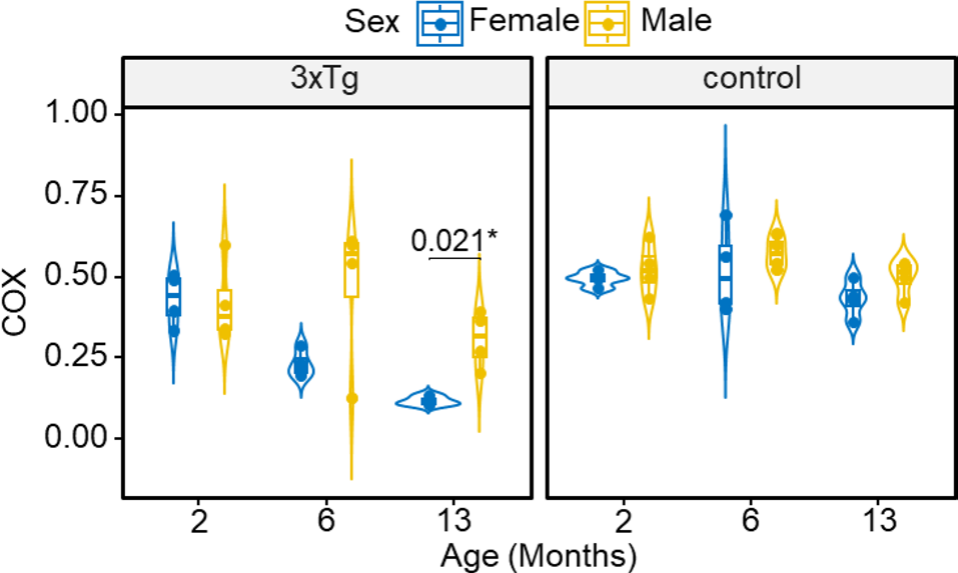
Cytochrome c oxidase (COX) activity in 3xTg vs. control mice. The activity of COX was in hypothalamic mitochondria from 3xTg female and male mice, alongside their control counterparts, at 2, 6, and 13 months of age. Statistical significance is indicated by asterisks, with results shown as mean ± SD of *n* = 5 per group (*P ≤ 0.05, **P ≤ 0.01, and ***P ≤ 0.001). At 13 months, COX activity was significantly reduced in females 3xTg relative to males

In an effort to investigate how oxidative phosphorylation (OXPHOS) machinery is affected by sex and age during normal aging and the progression of AD, we examined several mitochondrial-associated proteins in the hypothalamus. We conducted immunoblotting and relative quantification for protein levels for the subunits of OXPHOS complexes I through V, with normalization to total protein content (Fig. 3A-F). The immunoblotting results indicated that there were significant increases in the level of OXPHOS protein subunits specifically within complexes II, III, and V in the hypothalamus of 3xTg mice (Fig. 3B, C, E). The expression of complex II was significantly reduced in female 3xTg mice compared to males. No sex-based differences were found in control mice across any age group (Fig. 3B). Complex III expression varied significantly by sex, with female 3xTg mice displaying higher levels at 2 and 6 months, whereas control mice showed an increase in this protein only at 13 months (Fig. 3C). No significant differences in complex V expression were observed in the 3xTg mice. However, at 2 months of age, control mice exhibited sex-based differences, with higher levels in females, while no notable differences were found in other age groups (Fig. 3E). Furthermore, our analysis revealed that there were no statistically significant variations in the levels of protein subunits for complexes I and IV, irrespective of genotype or sex, across all age groups examined (Fig. 3A and D) (Fig. 4).

### Cytochrome C oxidase (COX) activity

Mitochondrial function was evaluated by measuring the enzymatic activity of COX in both female and male 3xTg mice and age-matched control mice. At 13 months of age, female 3xTg mice exhibited significantly lower COX activity compared to males (Fig. 4). No significant differences in COX activity were observed in control mice with respect to age or sex.

### Inflammatory proteins

**Fig. 5:**
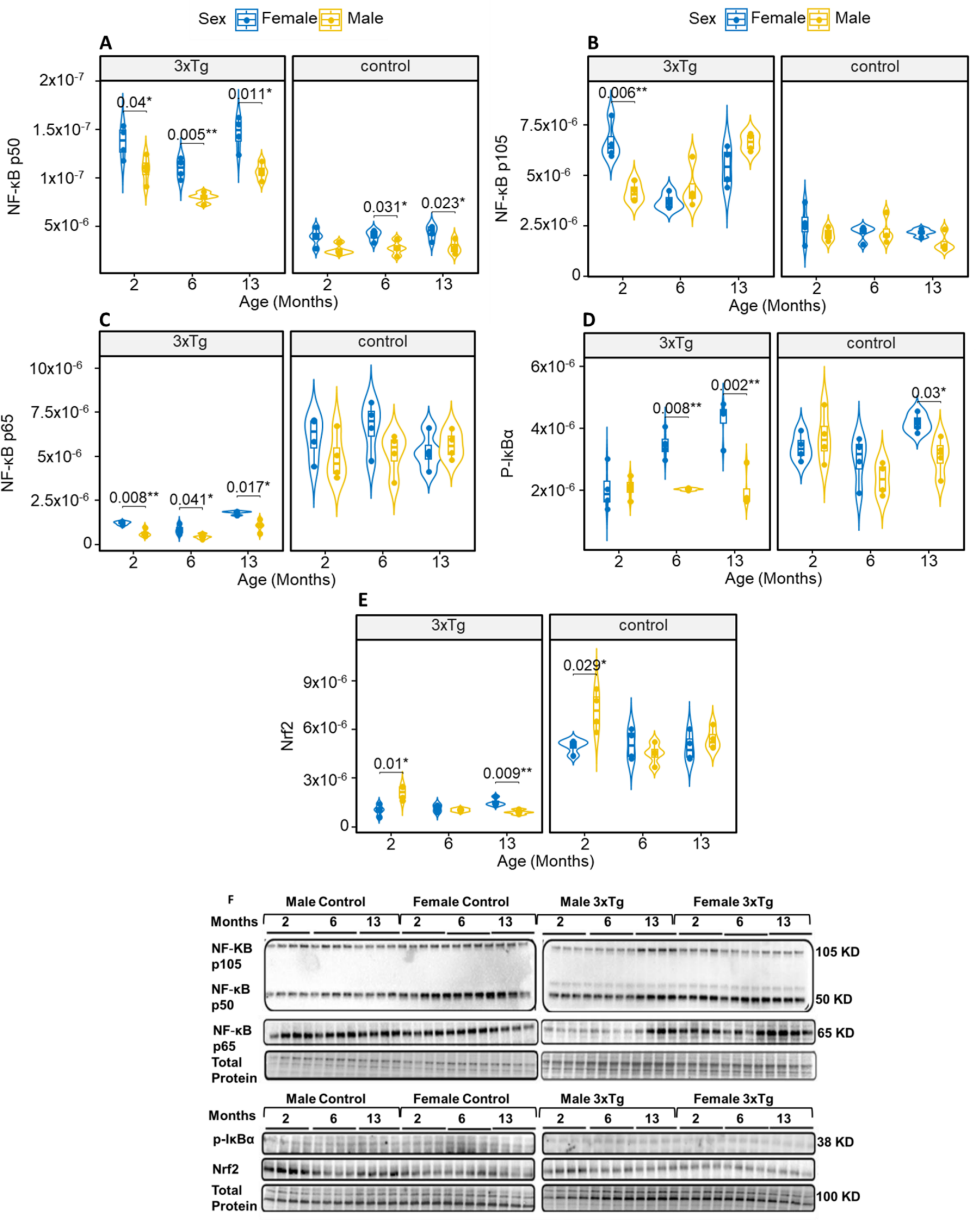
Levels of NF-κB subunits, pIκB-α, and Nrf2 in the hypothalamus of 3xTg mice vs. control mice. Western blot analysis shows the levels of NF-κB subunits (p50 (**A**), p105 (**B**), p65(**C**), pIκB-α (**D**), Nrf2 (**E**) in hypothalamic tissues from 3xTg female and male mice compared to their control counterparts at 2, 6, and 13 months of age. Quantification of these proteins was normalized to total protein (**F**). Asterisks indicate statistical significance compared to corresponding control groups. Results are presented as mean ± SD of *n* = 4 per group with significance levels marked as *P ≤ 0.05, **P ≤ 0.01, *** *P* ≤ 0.001, and ***P ≤ 0.001, and analyzed by three-way ANOVA. Significant sex differences in NF-κB and Nrf2 protein levels were observed in 3xTg mice across various ages. NF-κB p50 levels were elevated in females compared to males in all age groups (**A**). For NF-κB p105, females exhibited higher levels than males at 2 months, but no differences were found in older mice (**B**). NF-κB p65 showed reduced levels in male 3xTg mice across all ages, with no differences in control mice (**C**). Elevated pIκB-α levels were noted in females at 6 and 13 months, while control males only showed a reduction at 13 months (**D**). Nrf2 levels were downregulated in females at 2 months but increased in female 3xTg mice by 13 months, with control levels remaining stable (**E**)

To examine the presence of potential age-related sex differences on transcriptional regulation during normal aging and AD, immunoblots of several key transcription factor proteins or their subunits (NF-κB p50, p105, p65, pIκB-α, and Nrf2) implicated in neuroinflammatory signaling were measured in isolated hypothalamic mitochondria from 3xTg female and male mice vs. their respective controls at 2, 6, and 13 months of age (Fig. 5A-E). Relative levels of NF-κB subunits and Nrf2 (Fig. 5A-C, upper panel) were normalized to total protein (Fig. 5A-C, lower panel). Significant sex differences were detected in 3xTg NF-κB p50 in all age groups, where levels were elevated in females vs. males (Fig. 5A). We noted similar findings in control mice, but these were only evident at 6 and 13 months of age. Protein levels of NF-κB p105 were significantly different as a function of sex in 2 months old 3xTg mice, where levels were elevated in females vs. males (Fig. 5B). This increase was not sustained at later stages, as we found no sex differences in protein level of NF-κB p105 in older 3xTg female mice (Fig. 5B). Also, no significant sex differences in NF-κB p105 levels were observed in control mice across any age group (Fig. 5B). Protein levels of NF-κB p65 differed significantly by sex in all age groups studied, showing a reduction in male 3xTg mice compared to female 3xTg mice (Fig. 5C). However, no significant sex differences in NF-κB p65 were found in control mice in any age group (Fig. 5C). Significant sex differences were detected in 3xTg pIκB-α at 6 and 13 months, where levels were elevated in females vs. males (Fig. 5D). The expression of pIκB-α decreased in control mice, with a reduction observed in males only at 13 months (Fig. 5D). Significant sex differences in Nrf2 levels were observed in both control and 3xTg mice at 2 months, with levels downregulated in females compared to males (Fig. 5E). However, the expression of Nrf2 increased in female 3xTg mice by 13 months, while levels in control mice remained unchanged at that age (Fig. 5E).

### Cytokine/Chemokine assay

**Fig. 6:**
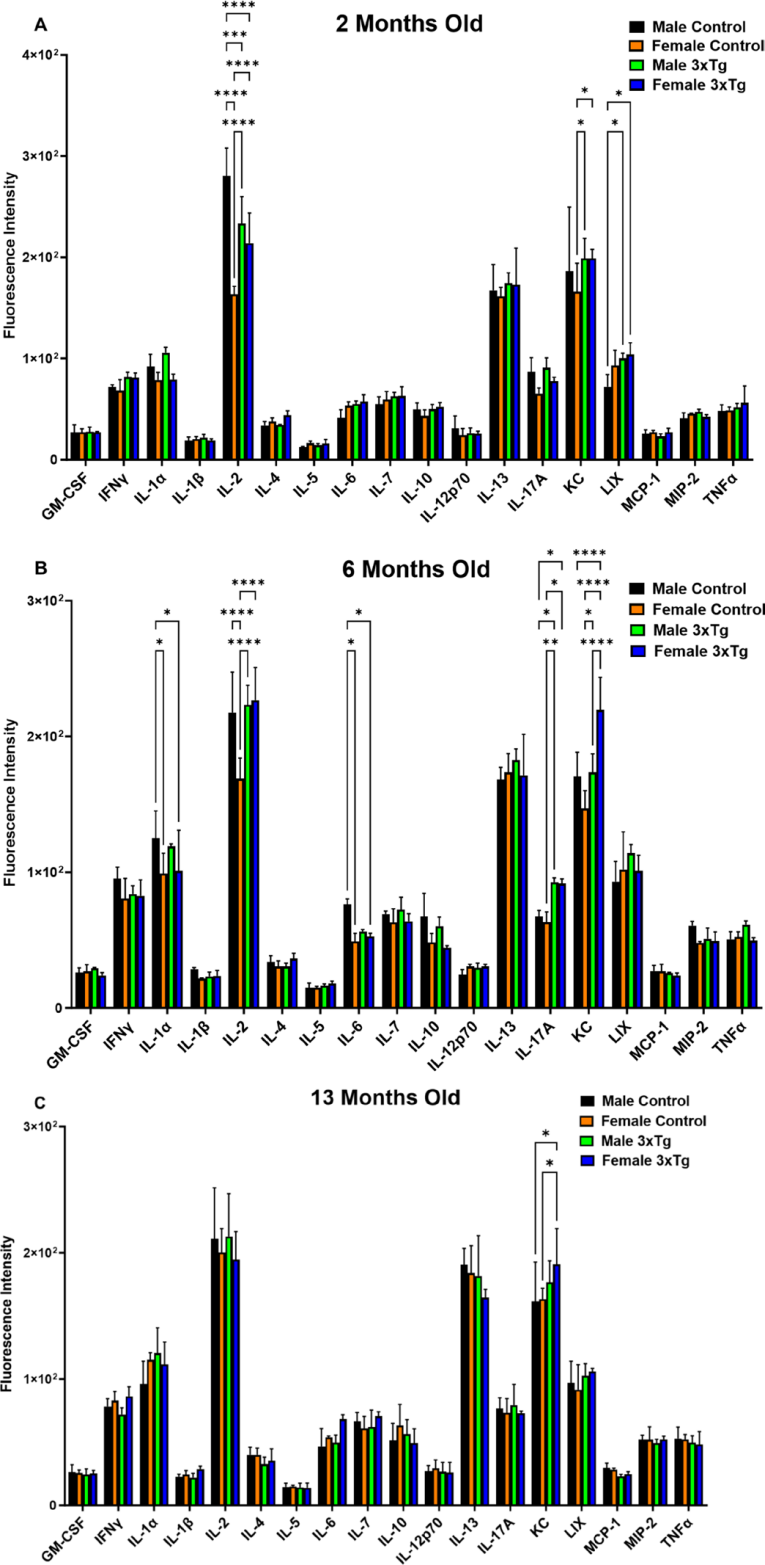
Levels of hypothalamic inflammatory biomarkers in 3xTg vs. control mice. Levels of GM-CSF, IFN-γ, IL-1α, IL- β, IL-2, IL-4, IL-5, IL-6, IL-7, IL-10, IL-12p70, IL-13, IL-17 A, KC, LIX, MCP-1, MIP-2, and TNF-α were assessed in hypothalamic tissues from both male and female 3xTg mice and their age-matched control counterparts at 2 (**A**), 6 (**B**), and 13 (**C**) months of age. Asterisks vs. corresponding control groups represent the statistical significance. Results are expressed as mean ± SD of *n* = 3 replicates (*P ≤ 0.05, **P ≤ 0.01, *** *P* ≤ 0.001, and ****P ≤ 0.0001) analyzed by two-way ANOVA. At 6 months of age, hypothalamic IL-1α levels were significantly lower in female controls compared to males. IL-2 levels were notably decreased in male 3xTg mice compared to male controls at 2 months of age. Conversely, female 3xTg mice exhibited a significant increase in IL-2 expression compared to female controls at both 2 and 6 months of age. Significant sex-based differences in IL-2 levels were observed only in the control group at these time points. At 6 months of age, IL-6 levels were significantly lower in female controls compared to males. No significant differences in IL-6 levels were found at 2 or 13 months of age. Female 3xTg mice showed a significant increase in IL-17 A levels compared to male controls, while male 3xTg mice also had significantly higher IL-17 A levels compared to their male controls. KC expression was higher in female 3xTg mice compared to female controls at 2, 6, and 13 months, with increased levels also observed in females relative to males at 6 months. LIX levels were significantly elevated in male 3xTg mice compared to male controls at 2 months. No significant differences in the expression levels of other inflammatory markers were observed based on genotype or sex in any of the age groups studied

Hypothalamic tissues were collected from both female and male control and 3xTg mice at 2, 6, and 13 months of age (Fig. 6A-C). These samples were homogenized and analyzed for the concentrations of various cytokines/chemokines using the multiplex array (Eve Technologies, Calgary, Alberta, Canada). At 6 months of age, hypothalamic IL-1α levels were significantly lower in female controls compared to males. IL-2 levels were notably decreased in male 3xTg mice compared to male controls at 2 months of age. Conversely, female 3xTg mice exhibited a significant increase in IL-2 expression compared to female controls at both 2 and 6 months of age. Significant sex-based differences in IL-2 levels were observed only in the control group at these time points. No significant differences in IL-2 levels were found at 13 months of age. At 6 months of age, IL-6 levels were significantly lower in female controls compared to males. No significant differences in IL-6 level were found at 2 or 13 months of age. Female 3xTg mice showed a significant increase in IL-17 A levels compared to male controls, while male 3xTg mice also had significantly higher IL-17 A levels compared to their male controls. No significant differences in IL-17 A levels were found at 2 or 13 months of age. The expression of KC was found to be increased in the female 3xTg as compared to female controls at 2, 6, and 13 months of age. Also, sex-based differences were observed in KC expression, with higher levels in female 3xTg mice compared to males at 6 months. LIX levels were significantly elevated in male 3xTg mice compared to male controls at 2 months of age. No significant differences in LIX level were found at 6 or 13 months of age. No statistically significant differences, as a function of genotype or sex, were detected in expression levels of GM-CSF, IFN-γ, IL- β, IL-4, IL-5, IL-6, IL-7, IL-10, IL-12p70, IL-13, IL-17 A, MCP-1, MIP-2, and TNF-α in any age group (Fig. 6A-C).

## Discussion

Changes in hypothalamic structure and function in AD are increasingly recognized, particularly regarding how early alterations may impact energy metabolism. Evidence suggests that mitochondrial dysfunction and inflammation are key factors in the onset and progression of AD, potentially resulting in pathology, synaptic dysfunction, and neuronal damage [47, 48]. An increasing body of evidence suggests that sex differences may significantly influence the variations in AD prevalence including behaviour, cognitive performance, disease progression, prognosis, and underlying pathology [49–51]. For example, a previous study from our lab revealed sex differences in memory performance, showing that 3xTg females experienced memory impairments earlier than males [44]. In the current study, we observed sex-based differences in mitochondrial function. In particular, we found that mitochondrial dysfunction in female 3xTg mice occurs as early as 2 months of age, whereas males experience this dysfunction at a much later stage. Consistent with our findings, a recent study also identified sex-specific metabolic phenotypes in 3xTg mice. Notably, female 3xTg mice displayed metabolic disturbances opposite to those observed in males, characterized by increased body and fat mass as well as impaired glucose tolerance, while the study reported that although male and female 3xTg mice were metabolically similar at 3 months of age, by 6 months, male mice with AD exhibited decreased weight gain, lower adiposity, and reduced blood glucose levels after a glucose challenge, in contrast to the females [52]. Other studies have shown that Aβ load increases with age in 3xTg mice, beginning at 6–8 months, with females consistently exhibiting higher Aβ levels [53]. Furthermore, neuroimaging research has indicated that brain atrophy rates are higher in female AD patients than their male counterparts, correlating with Aβ and tau changes and ApoE4 allele status [54]. Research also shows that, at 12 months, females show more tau tangles in their brains than males [54]. Another study reported that glucose intolerance and Aβ pathology worsen with age in 3xTg mice, especially in females [55]. Further, sex differences in mitochondrial outcomes were noted in the rodent model of traumatic brain injury (TBI) [56]. Specifically, female TBI mice exhibited early impairment at 12 h after severe controlled cortical impact (CCI), whereas mitochondria from injured male mice showed impairment at 24 h post-CCI [56]. These findings indicate that mitochondria in male and female brains can exhibit distinct profiles, highlighting various functional and structural differences. Overall, early mitochondrial deficits in females could accelerate Aβ accumulation, leading to earlier cognitive impairments and increased tau tangles. This could suggest a connection between impaired mitochondrial function and the increased vulnerability of females to neurodegenerative pathology, implying that sex-specific biological factors may influence the timing and progression of mitochondrial impairment in AD. By highlighting these differences, particularly the sex-based disparities in mitochondrial dysfunction, our study advances the field by emphasizing the need for tailored research strategies and potential interventions that take sex into account in the context of neurodegenerative diseases.

Mitochondrial dysfunction can trigger and sustain inflammatory responses through interconnected pathways, making it a key factor in numerous diseases and conditions [42, 57]. For instance, mitochondrial dysfunction can lead to increased production of reactive oxygen species (ROS), activating inflammatory pathways [58]. Additionally, disrupted cellular metabolism due to mitochondrial malfunction alters metabolite balances and signaling pathways, such as NF-κB, which regulates the expression of numerous inflammatory genes [59]. The correlative connection between mitochondrial components and inflammation becomes evident by identifying various metabolic by-products and molecules such as damage-associated molecular patterns (DAMPs) [60]. These molecules include mtDNA, mitochondrial ROS, mitochondrial calcium, ATP, cytochrome c, TFAM, cardiolipin, and iron [61]. The release of these substances into the cytosol or extracellular space triggers inflammation, illustrating how mitochondrial dysfunction contributes to inflammatory responses. Proteomics, genomics, and bioinformatics analysis demonstrated the disruption of mitochondrial oxidative phosphorylation machinery as hallmarks of AD [62, 63]. Additionally, the incidence of AD is higher in women compared to men, underscoring the need for further research into sex-specific differences in the development of the disease.

The current study examined mitochondrial bioenergetic profiles, along with levels of OXPHOS proteins, and COX activity in male and female control and 3xTg mice during normal aging and AD progression. We observed significant reductions in basal respiration, coupled respiration, maximal respiration, and spare respiratory capacity in female 3xTg mice compared to controls as early as 2 months of age. In contrast, declines in mitochondrial bioenergetic parameters in males were not observed until 6 months of age. Early metabolic deficits in mitochondrial function, particularly observed in females, suggest that there may be sex-specific differences in how mitochondrial function develops or is maintained during the early stages of life. Another study shows that alterations in hypothalamic bioenergetics may be attributed to reduced energy demands, potentially stemming from decreased hypothalamic neurogenesis reported in 3xTg mice [64].

In alignment with our OCR findings, we found significant differences in the expression of mitochondrial complex II and III in 3xTg mice. It has been suggested that levels of ETC subunit proteins can serve as indicators of OXPHOS, and therefore also serve as an index of mitochondrial respiration. Other studies showed a decrease in complex II, which leads to decreased ATP content in the APP/PS1 mouse model [65, 66]. Human studies have also demonstrated that patients with mild cognitive impairment (MCI) and early AD exhibit changes in mitochondrial gene expression and function [67]. Another study demonstrated that mitochondrial genes related to complex I and II of the OXPHOS pathways are downregulated in both early and advanced stages of AD and PD which can substantially impair ATP production and mitochondrial respiration in synaptic regions [68–70]. This impairment contributes to the synaptic dysfunctions commonly associated with these diseases. We observed a trend of reduced COX activity in female 3xTg mice compared to males across all age groups. At 13 months, COX activity was significantly lower in female 3xTg mice than in their male counterparts. This sex-based difference suggests that the reduced COX function in the ETC may be more pronounced in females, potentially leading to a greater decline in mitochondrial respiration and more severe disruptions in energy metabolism. In line with our findings, a study on Parkinson’s disease (PD) has also reported sex-specific effects on COX in astrocytes [71]. Another study has shown deficiencies in both Complex I and COX, in the substantia nigra neurons of patients with PD [72]. They proposed that COX deficiency appears only in the presence of Complex I deficiency and that this specific molecular pattern might be linked to changes in mitochondrial DNA (mtDNA) [72]. Also, another research has shown that a reduction in mtDNA content in the skeletal muscle of women at the onset of menopause may provide evidence for sexual dimorphism in bioenergetic and metabolic processes [73]. Quantitative proteomics studies have revealed significant alterations in mitochondrial proteomes, redox proteins, ATP synthase, and COX in females [74]. These findings suggest that females may be at greater risk for AD progression compared to men, potentially due to their distinct metabolic profiles and hormonal influences that affect mitochondrial function and oxidative stress responses.

Metabolic signals originating from mitochondria can regulate the inflammatory response [75]. In both normal aging and neurodegenerative diseases, certain inflammatory factors can be broadly neurotoxic. These responses are driven by positive feedback loops between microglia and astrocytes. When neurons are damaged, they release ATP and other DAMPs that activate glial cells, leading to the further release of inflammatory factors by astrocytes [76]. This cycle exacerbates the neurotoxic environment, accelerating neuronal damage and disease progression.

Inflammaging is a persistent inflammatory state associated with neurodegenerative diseases and cognitive decline in older adults [3, 6, 7]. Additionally, sex-specific variations in inflammasome proteins have been observed in different disease models [22, 34, 35]. However, a comprehensive comparison of inflammaging between male and female subjects has been missing.

In this study, we explored how sex and age influence the expression of transcription factors, inflammation-related proteins, cytokines, and chemokines in the hypothalamic tissue of both young and aged male and female 3xTg mice, as well as controls, throughout normal aging and neurodegeneration. One of the transcriptional factors we measured was NF-κB, a central inflammatory regulator with diverse effects upon activation. NF-κB can exert neuroprotective effects in neurons while promoting inflammatory responses in glial cells [77]. By assessing NFκB expression, we aimed to understand its dual role in modulating inflammation and its impact on normal aging and neurodegeneration in our AD mouse model. To this end, we found a robust upregulation of NF-κB p50 and 105 in the 3xTg as compared to controls, which could contribute to a greater inflammatory response and/or more rapid disease progression. Interestingly, the overexpression of NF-κB p50 occurred in a sex-dependent manner. NF-κB is known to regulate energy metabolism by balancing glycolysis and mitochondrial respiration [23]. In neurodegenerative diseases, its activation can be triggered by a cascade of signals from damaged neurons and glial cells. Confocal studies from our group and others have confirmed elevated levels of NF-κB p50 in AD astroglia compared to controls [24, 78]. Additionally, human studies have also demonstrated increased NF-κB p50 levels in the cerebral cortex of individuals with AD [79]. Another human study reported an increase in the synthesis of the NF-κB precursor, p105, along with the activation of NF-κB in AD brains [80]. Further, we found a reduced level of NF-κB p65 in 3xTg compared to controls, indicating widespread alterations in this AD model that are sex-independent. This reduction in NF-κB p65 is significant as it can influence the expression of mitochondrial genes critical for energy production and cellular function. According to Cogswell et al. (2003), the NF-κB pathway can regulate mitochondrial gene expression, including the COX III subunit, which is essential for complex IV of the mitochondrial ETC [25]. Additional research has highlighted the role of NF-κB in regulating various mitochondrial genes, including COX I and cytochrome b [81]. Specifically, studies have shown that the NF-κB p65 subunit can reduce levels of mtDNA-encoded cytochrome b mRNA in human cells in the absence of p53 [82]. Collectively, these findings suggest that NF-κB signaling plays a crucial role in modulating the enzymatic activity of respiratory ETC complexes. Interestingly, we found that female 3xTg mice exhibited higher levels of NF-κB subunits at a later stage compared to male 3xTg. This sex-based difference in NF-κB subunit levels was possibly associated with more pronounced neuroinflammation in females. Additionally, we found downregulation of the NF-κB inhibitor, IκB, in 3xTg mice which might be a regulatory mechanism to maintain balanced NF-κB activity. We observed a significant downregulation of the pIκB-α in 3xTg, which corresponds with decreased activity of the NF-κB p65 subunit. The decreases might reflect a compensatory or initially protective response to AD. Similar to NF-κB p65, pIκB-α levels were found to be significantly lower as a function of sex at 6 and 13 months of age. These findings highlight potential sex-specific variations in the inflammatory response in AD.

Another transcription factor we examined was Nrf2, which plays a crucial role in neurodegeneration and interacts with NF-κB [28]. In this study, we observed sex differences in Nrf2 expression in the 3xTg and control groups at 2 months of age, where females showed a reduction. In contrast, at 13 months of age, 3xTg females exhibit higher Nrf2 levels compared to males. Accumulating evidence on AD, PD, Huntington’s disease (HD), multiple sclerosis (MS), and amyotrophic lateral sclerosis (ALS) have demonstrated that the loss of Nrf2 exacerbates neurodegenerative symptoms [83]. This exacerbation includes elevated ROS, increased inflammation, decreased OXPHOS, synaptic plasticity, memory function, and proteotoxicity, all of which negatively impact neuronal and glial function and viability. Previous research has reported contrasting expression patterns between NF-κB p65 and Nrf2 [84, 85]. In contrast, our findings show decreases in hypothalamic NF-κB p65 and Nrf2 levels. Our results suggest a dynamic regulation of NF-κB p65 and Nrf2, where these transcription factors exhibit similar expression patterns under physiological and pathological conditions.

Several lines of research demonstrate that inflammation not only plays a critical role in accelerating the progression of neurodegenerative diseases, but also serves as a key initiator of these conditions. Both male and female immune responses undergo significant changes throughout the lifespan [19]. We observed a significant increase in chemokine KC (Keratinocyte-derived Chemokine, also known as C-X-C motif chemokine 1 (CXCL1) in female 3xTg mice compared to female controls at 2, 6, and 13 months of age. We also observed that the differences in KC chemokine expression at 6 months are sex-dependent, with higher levels found in female 3xTg mice compared to 3xTg males. Another study found that the expression of this chemokine is markedly increased in APP/PS1 transgenic mice. Additionally, treatment with an anti-CXCL1 antibody significantly impairs the accumulation of CD11b + CD45hi cells recruited from peripheral blood in the brains of these APP/PS1 mice. These results suggest that elevated CXCL1 levels in monocytes might contribute to the heightened presence of these cells in the brains of transgenic mice with AD. Interestingly, CXCL1 has been shown to facilitate the migration of monocytes from the bloodstream into the brain in AD, particularly in response to Aβ deposition [86]. This process can intensify inflammation and may play a role in accelerating the progression of AD.

Our analysis also showed that there was an increased upregulation of LIX (Lymphotactin, also known as CCL1) in 3xTg groups compared to controls at 2 months of age. There is substantial evidence linking disruptions in CXCL1 signaling to the pathomechanism of AD. For example, a previous study showed an increase in CCL1 mRNA and protein expression in the brains of APP/PS1 mice vs. controls [87]. Monocytes collected from AD patients overexpress CXCL1. When human brain microvascular endothelial cells are treated with Aβ, they show increased expression of CXCR2 and this interaction with CXCL1 contributes to the disruption of the blood-brain barrier (BBB) and enhances the migration of monocytes into the brain in AD patients [86]. Likewise, CXCL1 was found to trigger the phosphorylation of tau protein, leading to the formation of varicosities or bead-like structures along the neurites. This tau cleavage was also observed after intrahippocampal microinjection of lentiviral CXCL1 in aged mice [88]. Importantly, the expression of chemokines such as CXCL1 and CCL1 is modulated by NF-κB in many diseases such as cancer, diabetes, and neurological disorders [89–93]. Therefore, it is suggested that the overexpression of CXCL1 and CCL1 in AD may further drive NF-κB activation by intensifying inflammation, establishing a feedback loop that could worsen the inflammatory response. Our analysis showed that there were sex-based differences with IL-2 levels being lower in female controls than in males at 2 and 6 months of age. Additionally, the expression of this interleukin decreased in male 3xTg compared to male controls at these time points.

These findings align with earlier observations of reduced IL-2 levels in hippocampal tissues derived from AD patients and APP/PS1 mice [94, 95]. These findings suggested that a decline in IL-2 is linked to a diminished immune response. Other investigations have shown that IL-2 knockout mice experience difficulties in learning and memory formation, along with altered hippocampal development [96]. Other investigations show that IL-2 treatment improves AD pathology in APP/PS1 mice by enhancing Treg activation and increasing astrocytic activity, which helps clear Aβ and reduce plaque formation. It also reduces the Aβ42/Aβ40 ratio, potentially protecting neurons [97]. In APP/PS1 mice, IL-2 activates microglia, further aiding in disease management [98]. IL-2 knockout mice exhibit learning and memory deficits and altered hippocampal development [99]. Another study reported that IL-2 treatment in APP/PS1 mice supports recovery from memory deficits by promoting brain tissue remodelling, including improved synaptic plasticity [36]. These findings suggest an association among inflammation, Aβ pathology, and cognitive functions.

In our study, we observed a decrease in IL-1α and IL-6 levels specifically in 6-month-old female 3xTg mice and controls, with no such change in males. This finding contrasts with clinical studies, which report increased levels of these interleukins in the brains of patients with MCI and AD [100, 101]. The reduction in IL-1α and IL-6 in females may reflect a compensatory or initial neuroprotective response by the affected cells. This altered balance in IL-1α could suggest unique regulatory mechanisms or feedback responses in females, potentially influencing the neuroinflammatory environment.

At 6 months of age, female and male 3xTg mice showed a significant increase in IL-17 A levels compared to their prospective controls. This finding aligns with other studies that report elevated IL-17 A levels in the APP/PS1 mouse model. These studies also show that increased IL-17 A enhances TNF-α secretion by microglia through the NF-κB signaling pathway, thereby worsening neuroinflammation [102, 103]. Pre-clinical and clinical data suggest that IL-17 A promotes the formation of Aβ plaques and tau, impairs microglial phagocytosis, and triggers cognitive and synaptic deficits in both AD models and patients [39, 102]. Further, inhibiting IL-17 A has been shown to reduce AD pathology markers in the brains of AD patients, mitigate neuroinflammation, and slow the progression of the disease. These repeated observations of elevated IL-17 across various models and studies underscore its potential importance as a target for both understanding and potentially modulating AD progression and associated mechanisms.

Overall, the distinctive sex-based variations in cytokine and chemokine profiles observed throughout normal aging and across the stages of AD (early, middle, and late) highlight the potential of these factors as insightful biomarkers for monitoring disease progression and development.

## Conclusions

As we age, the hypothalamus, vital for regulating metabolism and maintaining balance, experiences significant changes. Early mitochondrial dysfunction and chronic inflammation in this region disrupt its normal function, which, coupled with the broader metabolic shifts and inflammatory processes of aging, plays a crucial role in the development and progression of AD. This study indicates that females may experience more pronounced mitochondrial and metabolic changes early in the disease, along with sex-based differences in inflammation during AD progression. Integrating sex and age into AD research is essential for accurately assessing potential treatments and improving the relevance of preclinical results. This approach ensures a more comprehensive understanding of AD and supports the development of personalized therapies, ultimately enhancing patient care by tailoring treatments to individual needs and improving overall outcomes.

## Data Availability

No datasets were generated or analysed during the current study.
